# Hurricanes as an enabler of Amazon fires

**DOI:** 10.1038/s41598-021-96420-6

**Published:** 2021-08-20

**Authors:** Enoch Yan Lok Tsui, Ralf Toumi

**Affiliations:** 1 Department of Physics, Imperial College London, London, SW7 2AZ UK; 2 Leverhulme Centre for Wildfires, Environment and Society, London, UK

**Keywords:** Atmospheric dynamics, Natural hazards

## Abstract

A teleconnection between North Atlantic tropical storms and Amazon fires is investigated as a possible case of compound remote extreme events. The seasonal cycles of the storms and fires are in phase with a maximum around September and have significant inter-annual correlation. Years of high Amazon fire activity are associated with atmospheric conditions over the Atlantic which favour tropical cyclones. We propose that anomalous precipitation and latent heating in the Caribbean, partly caused by tropical storms, leads to a thermal circulation response which creates anomalous subsidence and enhances surface solar heating over the Amazon. The Caribbean storms and precipitation anomalies could thus promote favourable atmospheric conditions for Amazon fire.

## Introduction

South America, together with Equatorial Asia, are the two major regions contributing to the global net fire carbon emission^1^. High-frequency fires have also been shown to impact the unique ecological structure and biodiversity in the Amazon^2^. The drivers of fires in the Amazon region have gained much interest^3–5^. It is well known that Amazonian fires are ignited by humans^6–8^ and are thus not purely meteorological events. In this paper, the meteorological conditions that enable fires and are thus a cause of interannual variability are the principal concern. Chen et al.^4^ proposed an empirical seasonal prediction model for the total fire count from May onwards using North Atlantic sea surface temperature (SST) during the boreal spring. Fernandes et al.^3^ studied the relationships among West Amazon fires and precipitation ($76^{\circ }\,\,\mathrm {W}$–$70^{\circ }\,\,\mathrm {W}$, $14^{\circ }\,\,\mathrm {S}$–$3^{\circ }\,\,\mathrm {S}$) from July to September and North Atlantic SST in the preceding months. These predictions are based on the prevailing understanding that anomalously high tropical North Atlantic SST relative to the tropical South Atlantic results in a northwards shift of the Atlantic Intertropical Convergence Zone (ITCZ)^9,10^ and thus reduces the amount of precipitation the Amazon receives^11–13^. Yoon and Zeng^12^ showed that the North Atlantic SST correlates with the mean precipitation of the whole Amazon catchment throughout the year, except in the height of the boreal winter. However this area is much larger than the active fire region. Reduced Amazon precipitation is thought to promote fire activities there^3,4,14^. These studies have focused on seasonal prediction and still leave open the question on the direct physical link between the Atlantic and Amazon fires during the actual fire season. Here we identify this link as through anomalous precipitation in the ITCZ and in particular, for the first time, the important role of Caribbean storms.

**Figure 1:**
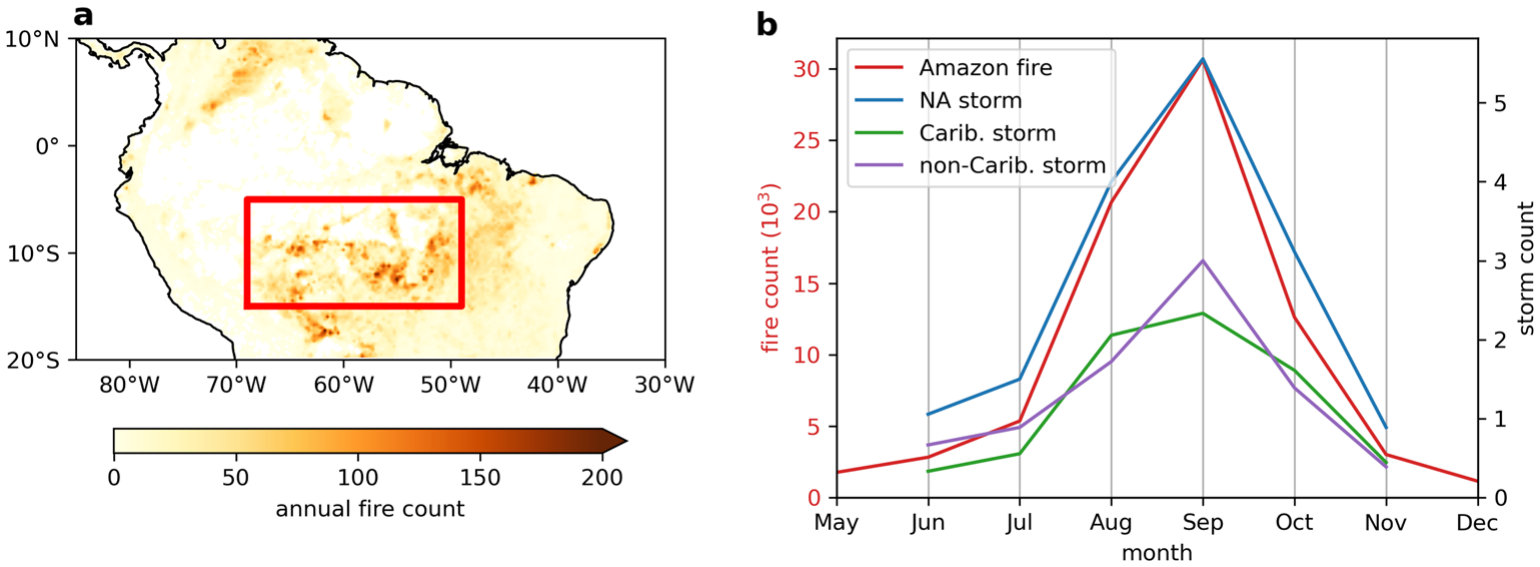
Climatology of Amazon fires and North Atlantic tropical storms. (**a**): Average annual fire count between 2001 and 2018. The red box is the most fire-active region. (**b**): Seasonal cycles of Amazon fire count and North Atlantic (NA), Caribbean (Carib.) and non-Caribbean (non-Carib.) tropical storm number computed from 2001 to 2018.

**Figure 2:**
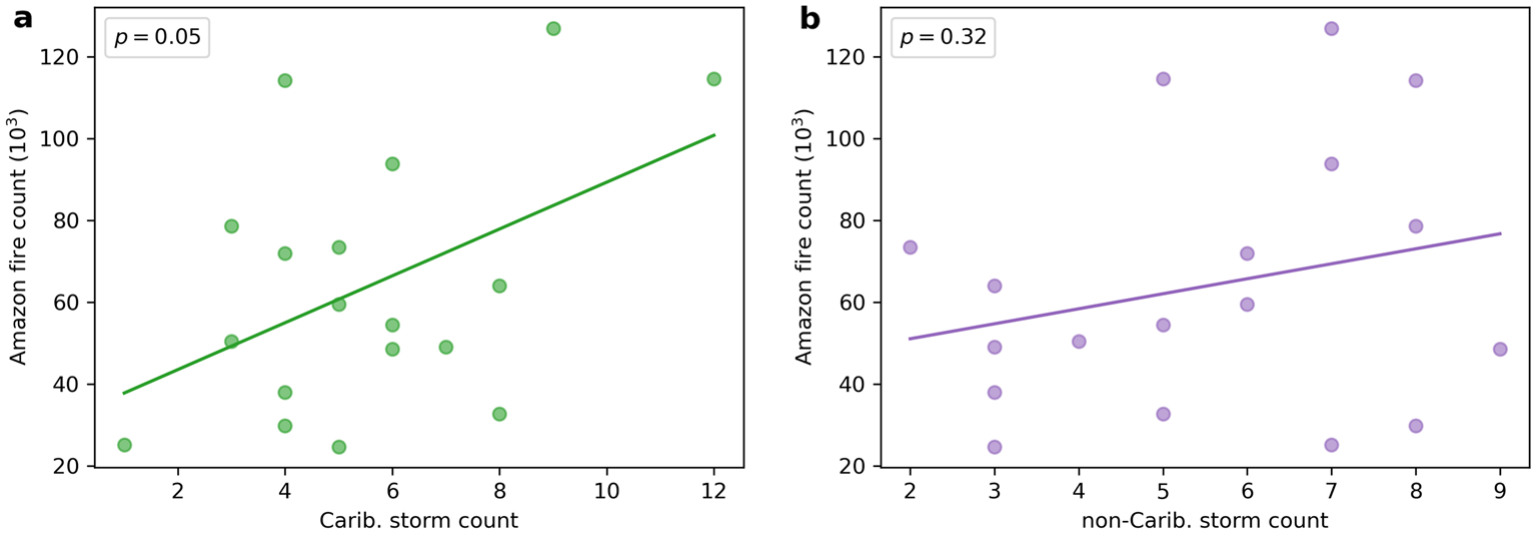
Relationship between Amazon fires and North Atlantic tropical storms. (**a**): Scatter plot with linear best fit of ASO Amazon fire count against ASO Caribbean tropical storm count in the years 2001–2018. (**b**): Same as (**a**) except for non-Caribbean tropical storm count.

**Figure 3:**
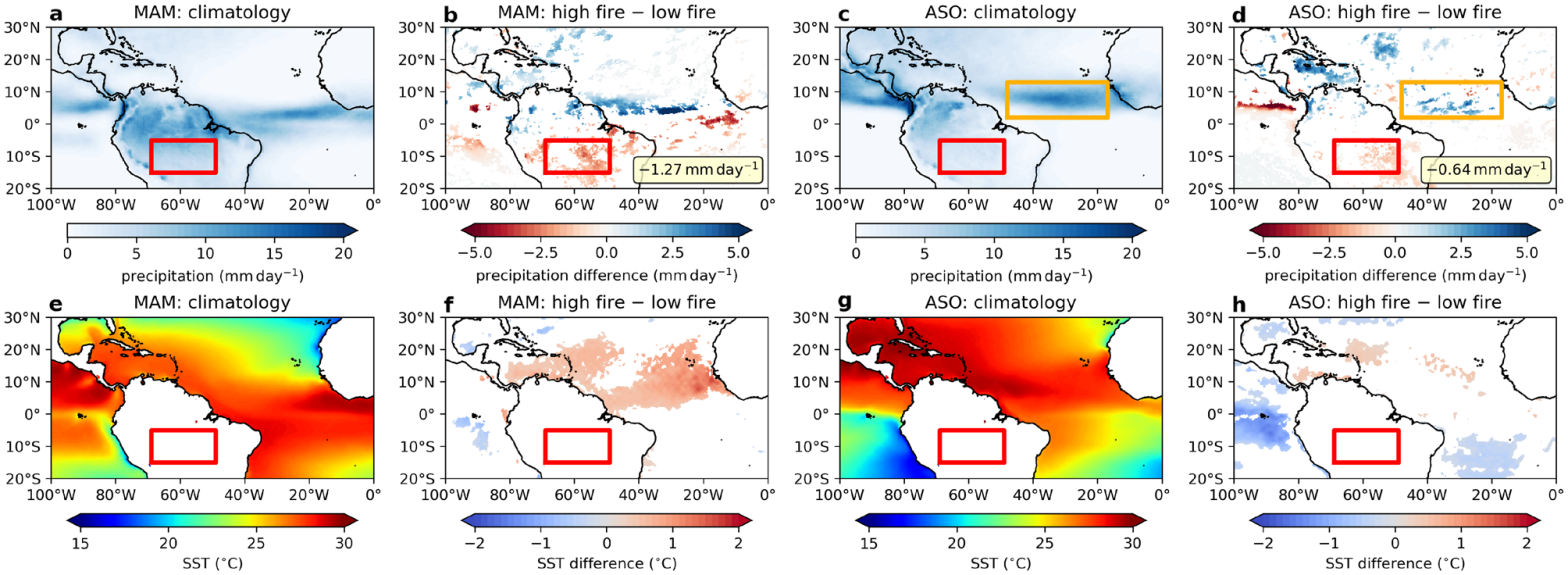
Precipitation and SST climatology and anomalies. (**a**), (**c**): Climatology of (**a**) MAM and (**c**) ASO precipitation between 2001 and 2018. (**b**), (**d**): Same as (**a**) and (**c**) except for composite difference (significant at $95\%$ confidence interval) of the top and bottom 5 years between 2001 and 2018 ranked by ASO Amazon fire count. Average precipitation difference over the Amazon is given in the lower right. (**e**), (**g**): Same as (**a**) and (**c**) except for SST. (**f**), (**h**): Same as (**b**) and (**d**) except for SST. (**a**)–(**h**): The red box is the Amazon fire region. (**c**)–(**d**): The orange box includes the Atlantic ITCZ.

The SST affects tropical storm activities and properties. An increase in SST increases water vapour in the lower troposphere, both of which make more energy available for atmospheric convection and tropical cyclone development^15^. The intensity and the number of tropical cyclones in the North Atlantic could increase with increasing tropical North Atlantic SST^16,17^. Dynamical factors are also important as tropical cyclone genesis and intensification is favoured by weak vertical wind shear^18^, low-level cyclonic circulation^19^ and a weakening of the easterly trade wind over the Caribbean^20^.

**Figure 4:**
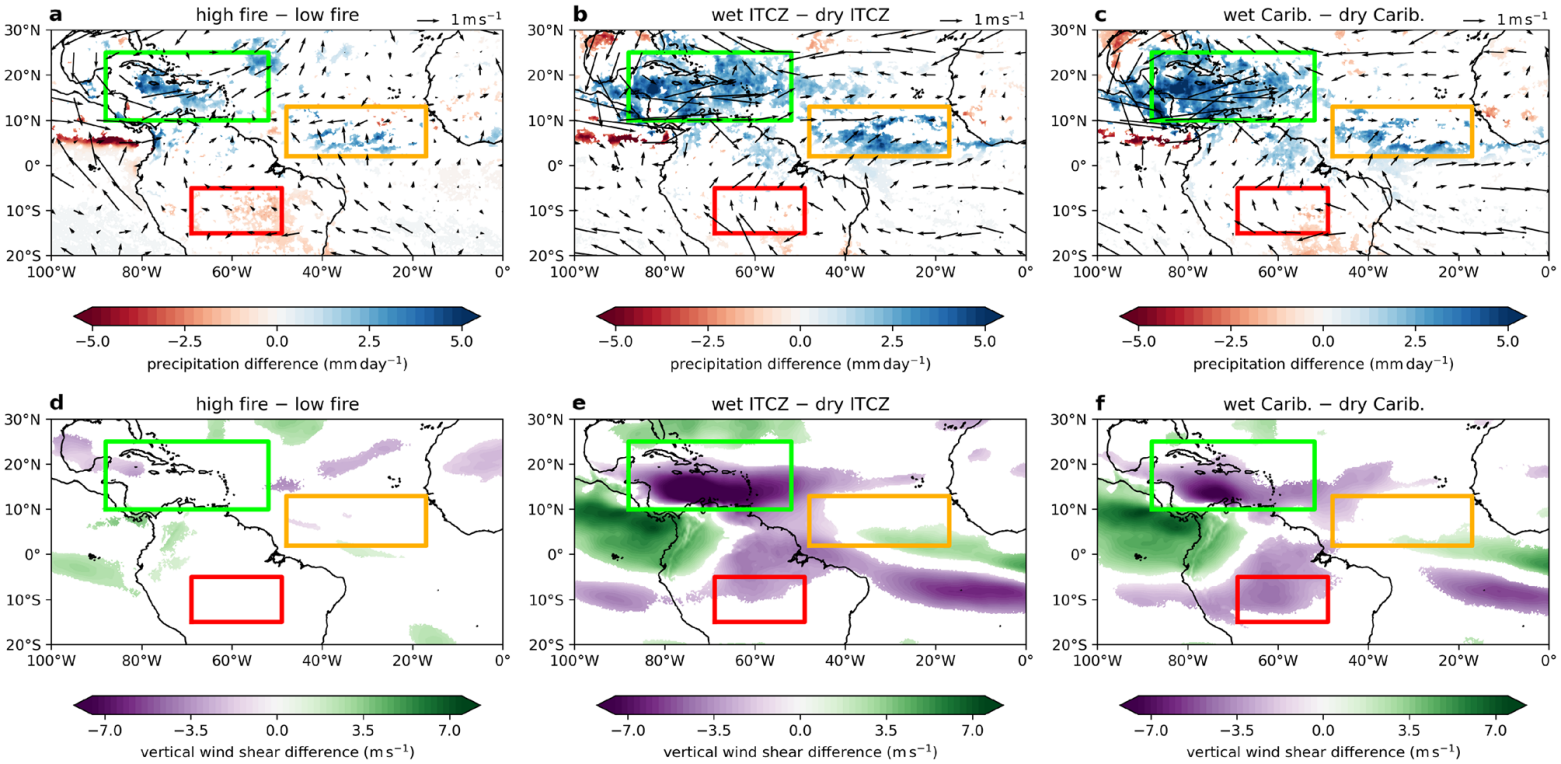
Horizontal wind, precipitation and vertical wind shear anomalies. (**a**)–(**c**): Composite difference for ASO of horizontal wind at $850\,\mathrm {hPa}$ and precipitation (significant at $95\%$ confidence interval) of the highest and lowest 5 years between 2001 and 2018 ranked by (**a**) ASO Amazon fire count, (**b**) ASO Atlantic ITCZ precipitation and (**c**) ASO Caribbean precipitation. (**d**)–(**f**): Same as (**a**)–(**c**) except for 850–200 hPa vertical wind shear. (**a**)–(**f**): The boxes are Amazon (red), Atlantic ITCZ (orange) and Caribbean (green).

**Figure 5:**
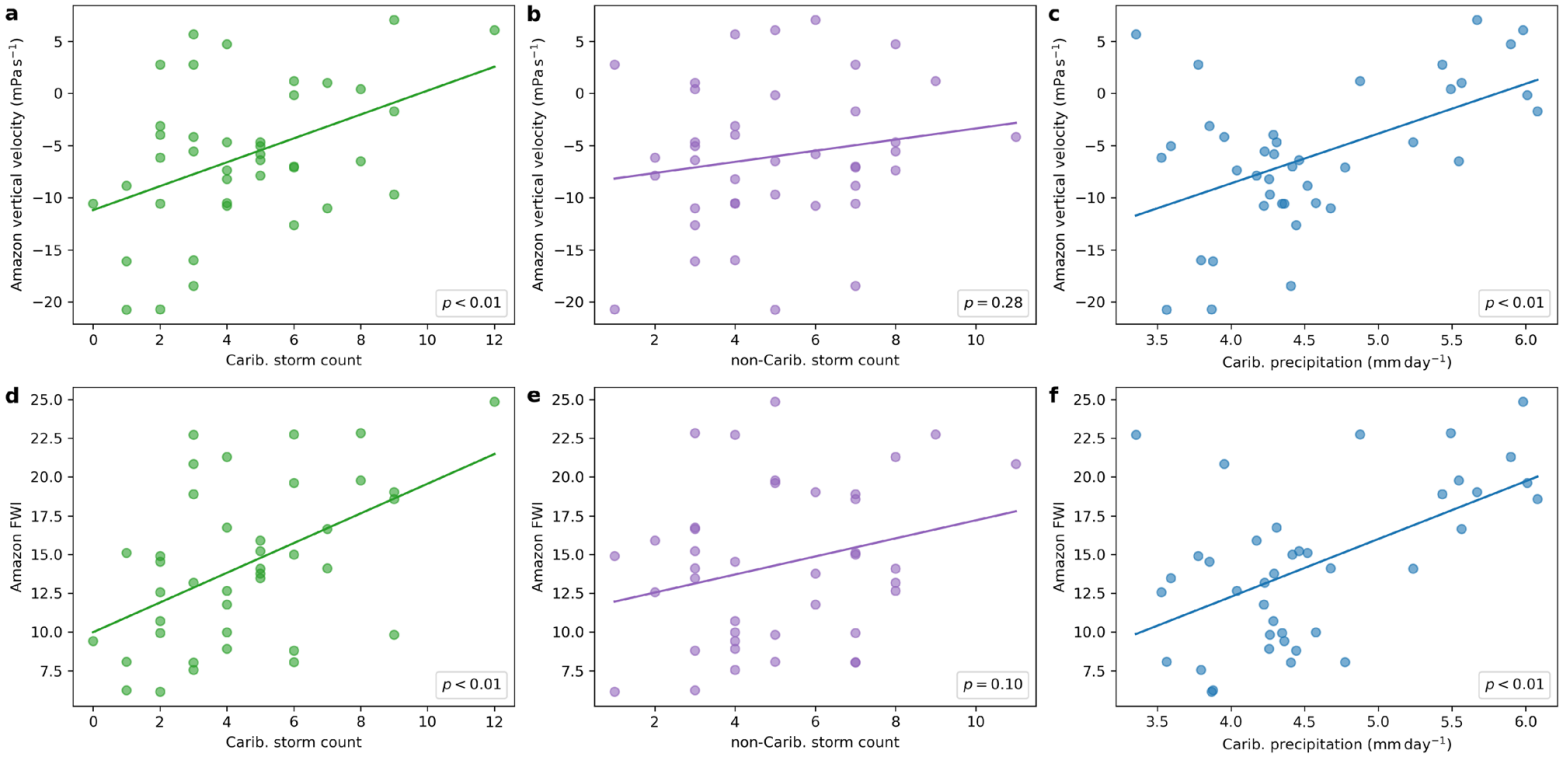
Relationship between the Amazon and the Caribbean. (**a**): Scatter plot with linear best fit of ASO Amazon vertical velocity at $600\,\mathrm {hPa}$ against ASO Caribbean tropical storm count computed from 1980 to 2019. (**b**): Same as (**a**) except for non-Caribbean tropical storm count. (**c**): Same as (**a**) except for Caribbean precipitation. (**d**)–(**f**): Same as (**a**)–(**c**) except for Amazon FWI.

**Figure 6:**
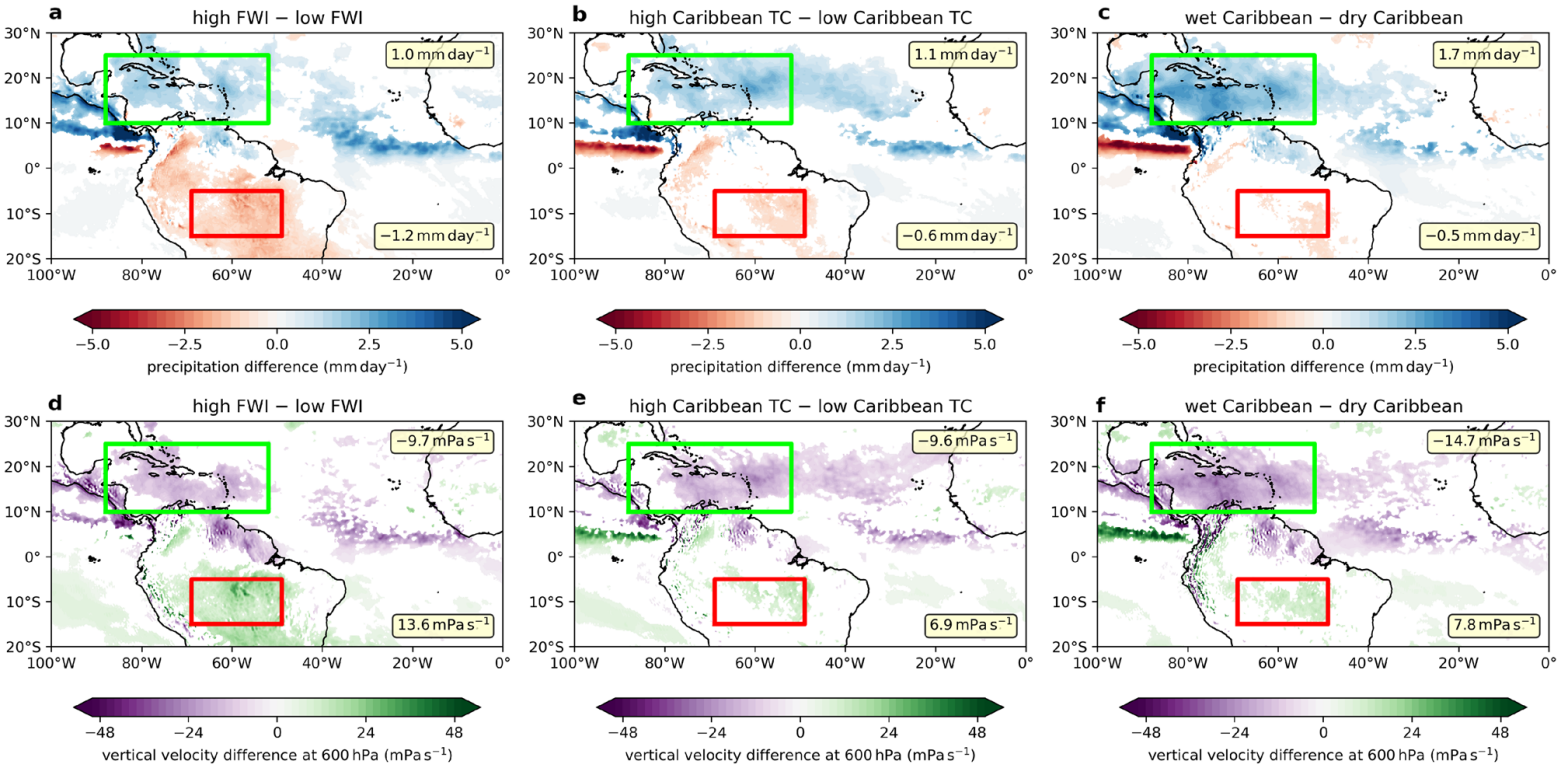
Thermal direct cell between the Caribbean and the Amazon. (**a**)–(**c**): Composite difference (significant at $95\%$ confidence interval) for ASO precipitation of the top and bottom 13 years between 1980 and 2019 ranked by (**a**) ASO Amazon FWI, (**b**) ASO Caribbean tropical storm count and (**c**) ASO Caribbean precipitation. Average precipitation difference (significant at $95\%$ confidence interval) over the Caribbean and the Amazon are given in the upper right and lower right respectively. (**d**)–(**f**): Same as (**a**)–(**c**) except for vertical velocity at $600\,\mathrm {hPa}$.

Chen et al.^14^ identified a relationship between June to November Amazon fires, and earlier (March to June) North Atlantic SST anomalies and the number of annual North Atlantic tropical cyclones. Intriguingly, they showed that the correlation between fires and tropical storms is actually greater than that between SST and either fires or tropical storms. They concluded that the correlation between fires and tropical storms cannot be fully explained by assuming only a common linkage of the two with the SST. However, they did not identify other possible causes or explanations for this. Here we hypothesise that tropical cyclones rainfall could play an active role in promoting Amazon fire conditions and the role of SST is actually minor. We suggest that anomalous precipitation and associated latent heating in the Caribbean due to tropical storms can provide a direct link between North Atlantic tropical storms and Amazon fires through a direct thermal circulation anomaly.

## Results

### Seasonal cycles of storms and fires

The annual fire count in the Northern South America is shown in Fig. 1a, from which the most fire-active region ($69^{\circ }\,\,\mathrm {W}$–$49^{\circ }\,\,\mathrm {W}$, $15^{\circ }\,\,\mathrm {S}$–$5^{\circ }\,\,\mathrm {S}$) in the Amazon is identified. Figure 1b shows the seasonal cycles of North Atlantic tropical storm number and Amazon fire count from 2001 to 2018. The seasonal cycles of storm and fire are in phase with a clear maximum in September. The 3 months from August to October (ASO) account for more than $75\%$ of North Atlantic tropical storm counts, Caribbean ($88^{\circ }\,\,\mathrm {W}$–$52^{\circ }\,\,\mathrm {W}$, $10^{\circ }\,\,\mathrm {N}$–$25^{\circ }\,\,\mathrm {N}$) storm counts, non-Caribbean storm counts (North Atlantic storms that did not enter the Caribbean box) and Amazon fire counts throughout the year. Figure 2 shows scatter plots with linear best fits for ASO Amazon fire count against both Caribbean and non-Caribbean ASO tropical storm number. Correlation analysis reveal significance between Amazon fire count and North Atlantic ($r = 0.58$, $p < 0.05$) and Caribbean ($r = 0.56$, $p < 0.05$) storm number but not with non-Caribbean storm number ($r = 0.13$, $p = 0.60$). We also performed correlation analysis for other 3-month windows between June and November (JJASON) and for JJASON. The results are similar to those for ASO, in particular across all windows analysed, the correlations between Amazon fire and tropical storm remain significant for North Atlantic (except for JJA) and Caribbean storms and insignificant for non-Caribbean storms. Motivated by the seasonal cycles of fire and storm, we focus on ASO, which is the most active season for both, for the rest of the paper.

### North Atlantic SST and ITCZ behaviour

Previous work was focused on the boreal spring and summer, which precedes the fire season. We find the behaviour of the variables in spring (MAM) quite different to ASO. We rank the years 2001–2018 by Amazon fire count and determined the composite anomalies of the precipitation and SST between the highest and lowest 5 years. In MAM, the fire region is immediately to the south of the ITCZ (Fig. 3a), thus any meridional shift of the ITCZ will directly affect the amount of precipitation the fire region receives. However, in ASO, the ITCZ has shifted much further north ($48^{\circ }\,\,\mathrm {W}$–$17^{\circ }\,\,\mathrm {W}$, $2^{\circ }\,\,\mathrm {N}$–$13^{\circ }\,\,\mathrm {N}$) and no longer overlaps the fire region (Fig. 3c). In MAM, there is a clear meridional shift of the Atlantic ITCZ between the high and low fire years (Fig. 3b). However, no such shift is observed in ASO rather it is the amount of precipitation that is different (Fig. 3d). The composite precipitation anomaly over the fire region is significant in both MAM and ASO, with the absolute magnitude during ASO being around half of that during MAM. Furthermore, the correlation of ASO fire count with Amazon precipitation is significant for both MAM ($r = -0.50$, $p < 0.05$) and ASO ($r = -0.55$, $p < 0.05$; non-detrended: $r = -0.40$, $p = 0.10$). A clear tropical North Atlantic SST warm anomaly is observed in MAM (Fig. 3f). However, this SST signal is barely significant in ASO (Fig. 3h). The SST climatology in these two seasons are shown in Fig. 3e, g. North Atlantic SST in MAM does appear to act as a precursor to the precipitations over parts of the North Atlantic in ASO. In particular, MAM North Atlantic SST correlates significantly with both ASO Atlantic ITCZ ($r = 0.85$, $p < 0.01$) and ASO Caribbean ($r = 0.77$, $p < 0.01$) precipitations.

### Atlantic ITCZ and Caribbean precipitation

We next explore the relationships between Amazon fire activities, ASO ITCZ precipitation, Caribbean precipitation and North Atlantic tropical cyclones through composite difference of anomalous years. We ranked the years 2001–2018 by three criteria: fire count, Atlantic ITCZ precipitation and Caribbean precipitation. The composite anomalies of the precipitation, horizontal wind at $850\,\mathrm {hPa}$ and 850–200 hPa vertical wind shear between the highest and lowest 5 years were determined. The anomaly patterns for the variables studied show common features across all three criteria. In direction consistent with more fires, there is more precipitation in the Caribbean and the ASO Atlantic ITCZ (Fig. 4). There are also anomalous low-level westerlies which are part of an extended anomalous low-level cyclonic circulation over the entire Caribbean. Reduced vertical wind sheer is found along a corridor where much of the North Atlantic tropical cyclones develop, although between the high and low fire years, a large part of this reduction is deemed insignificant by bootstrapping. These conditions of enhanced low level vorticity and reduced vertical wind shear favour tropical cyclone genesis and intensification. Anomalous convergence is found in the Atlantic ITCZ including strong inflow from the south driving enhanced precipitation. The precipitation over the Atlantic ITCZ and the Caribbean are highly correlated ($r = 0.84$, $p < 0.01$), showing coherence of these two regions.

### Caribbean storms and Amazon fires

We next explore the relationships between the Caribbean storm count and the fires. The fire count and the Caribbean precipitation ($r = 0.59$, $p < 0.01$; non-detrended: $r = 0.52$, $p < 0.05$), the Caribbean tropical storm number and the Caribbean precipitation ($r = 0.66$, $p < 0.01$) and the fire count and the Caribbean tropical storm number ($r = 0.56$, $p < 0.05$) all show significant correlations. The fire count also correlates with the Atlantic ITCZ precipitation ($r = 0.56$, $p < 0.05$; non-detrended: $r = 0.45$, $p = 0.06$) but not with non-Caribbean storm number ($r = 0.13$, $p = 0.60$).

If we hypothesise an active role for tropical cyclones, it is important to estimate the storm contribution to Caribbean precipitation anomalies. To test this, we replaced the precipitation of Caribbean tropical storm days with the *n*-day moving average non-storm day climatology. By taking the difference, we inferred that the tropical storms account for 35–36$\%$ (*n* between 3 and 15) of the precipitation anomaly between the wettest and driest 5 years in the Caribbean. This result is robust and larger ($65\%$) when we simply replace storm days with zero precipitation. Therefore, tropical cyclones contribute substantially to precipitation anomalies in the Caribbean. We can also examine individual year to support the relationship. For example, 2010 was a year with record (over the years studied here) Atlantic ITCZ precipitation, record Caribbean precipitation and record storm count. 2010 was also the year with the second highest Amazon fire count.

### Atmospheric Amazon fire risk factors

The connection between the Caribbean and the Amazon can be further explored by examining the tropical storms and precipitation over the Caribbean and the vertical velocity, surface solar radiation flux and a fire risk index (FWI) over the Amazon. While TRMM precipitation data and MODIS fire data are available only respectively from 1998 and 2001 onwards, ERA5 precipitation data and the FWI produced by the Copernicus Emergency Management Service for the European Forest Fire Information System from ERA5 data allows us to analyse a much longer period from 1980 to 2019. For the common period with both MODIS and FWI data, the fire count and risk index are positively correlated with significance ($r = 0.58$, $p < 0.05$; non-detrended: $r = 0.31$, $p = 0.21$). As with fire count, the FWI is significantly correlated to both Caribbean precipitation ($r = 0.58$, $p < 0.01$) and Caribbean storm number ($r = 0.33$, $p = 0.05$; non-detrended: $r = 0.50$, $p < 0.01$) but not with non-Caribbean storms ($r = 0.13$, $p = 0.41$) between 1980 and 2019. During the same period, the vertical velocity at $600\,\mathrm {hPa}$ shows significant correlations with the FWI ($r = 0.83$, $p < 0.01$), Caribbean precipitation ($r = 0.49$, $p < 0.01$) and Caribbean storm number ($r = 0.32$, $p < 0.05$; non-detrended: $r = 0.43$, $p < 0.01$) but not with non-Caribbean storms ($r = 0.08$, $p = 0.62$). Figure 5 shows that suppressed ascent and enhanced FWI over the Amazon is associated with more Caribbean storms and precipitation but not with non-Caribbean storms. The vertical velocity at $600\,\mathrm {hPa}$ is correlated with surface solar radiation flux ($r = 0.93$, $p < 0.01$), which is in turn correlated with the FWI ($r = 0.85$, $p < 0.01$). We further rank the years 1980–2019 by Amazon FWI, Caribbean tropical storm count and Caribbean precipitation and determined the composite anomalies of the precipitation and vertical velocity at $600\,\mathrm {hPa}$ between the highest and lowest 13 years. Figure 6 shows that when there is higher FWI in the Amazon, more precipitation and ascent over the Caribbean, there is also significant anomalous descent and reduced precipitation over the Amazon. The anomalous subsidence can reduce any local mean ascent. This in turn reduces the cloud cover and enhances the surface solar radiation flux. The FWI is enhanced by lower relative humidity and higher surface temperature.

## Discussion

We find that the seasonal cycles of North Atlantic tropical storms and South Amazon fires are in phase and both peak in September. Furthermore, most of the Amazon’s fires and North Atlantic tropical storms are found within ASO. This establishes ASO as a critical season for understanding a possible teleconnection between the two. The Amazon fire count is significantly correlated only with Caribbean tropical storm number ($r = 0.56$, $p < 0.05$) but not with the non-Caribbean ones ($r = 0.13$, $p = 0.60$), even though the Caribbean and non-Caribbean tropical storms both account for around half of all North Atlantic tropical storms on average. Correlation and composite analysis further reveal a link between Caribbean tropical storm count and Amazon fires via Caribbean and Amazon vertical atmospheric motion. Much of the ASO precipitation and ascent anomaly in the Caribbean can be accounted for by the tropical storms in the region. We further find anomalous atmospheric conditions favourable for North Atlantic tropical cyclone genesis and intensification when there are more fires. The ITCZ precipitation intensification is also associated with more Amazon fires.

The current paradigm links the fire variability to the varying amount of precipitation over the Amazon^3,4,14^. The Amazon precipitation variability is in turn attributed to meridional Atlantic ITCZ shifts responding to Atlantic SST anomalies^9,10^. These studies aimed to predict fire activity and thus were focused on precursor variables in the boreal spring and summer. Our study focuses exclusively on the peak fire season (ASO) and concurrent atmospheric conditions. We do not find a meridional shift in the ITCZ during this season rather anomalous precipitation over the Caribbean (and the ITCZ). These findings are consistent with current understanding of South America precipitation variability^21^, where the meridional shift of the Atlantic ITCZ is attributed to the precipitation variability in the boeral spring but not autumn. It is clear from these results that the Amazon fire count and precipitation in the peak fire season, ASO, have very different relationships with the Atlantic precipitation and SST than those studied previously for pre-seasons.

From our study a more complete understanding of the drivers of Amazon fires emerges and we identify possible sources of seasonal Amazon fire predictability. The MAM Atlantic SST anomalies could be a persistent precursor to an enhanced ASO Atlantic ITCZ, which favours tropical cyclones. This is supported by a significant correlation between the two ($r = 0.85, p < 0.01$). Here we conclude that it is the ASO Caribbean precipitation and tropical storms that can provide a plausible direct physical link to the fires. Another example of tropical cyclones and fires as a pair of remote compound extreme events with teleconnection in the zonal rather than meridional direction has recently been reported by Stuivenvolt Allen et al.^22^.

An active hurricane season in the Caribbean brings anomalous precipitation and therefore latent heating. Localised heating and ascent to the north of the Equator drives a thermally direct circulation causing raised pressure and anomalous subsidence in the Southern Hemisphere akin to the Hadley Cell circulation. This anomalous subsidence reduces precipitation and cloud cover, promotes surface solar heating and leads to higher surface temperatures which are favourable for fire.

In the classic Hadley Cell, warm rising air near the Equator reaches the stable tropopause and travels down the high-level pressure gradient polewards, cools and sinks in the subtropics. Studies have found that similar circulation can develop with an off-equatorial ascending branch to the north and a descending branch to the south of the Equator. Simulations by Rodwell and Hoskins^23^ incorporating orography and forced with heating of the monsoon precipitation showed that in both the Americas and West Africa, off-equatorial heating in the Northern Hemisphere induces local thermal, Hadley-type, circulation with an ascending branch around the heating region and a descending branch to the south across the Equator. Krishnan et al.^24^ simulated the Indian summer monsoon with a precipitation peak over the Bay of Bengal ($7^{\circ }\,\,\mathrm {N}$–$23^{\circ }\,\,\mathrm {N}$) and a thermal direct circulation with a descending branch between $35^{\circ }\,\,\mathrm {S}$ and $15^{\circ }\,\,\mathrm {S}$.

The thermal direct cell circulation is also captured by the simplified Matsuno–Gill model^25,26^. In one of the solutions^26^, the area of subsidence is equidistant from the Equator and zonally displaced slightly east of the heating region. In another idealised simulation^27^ with heating centred at $10^{\circ }\,\,\mathrm {N}$, subsidence is observed between $20^{\circ }\,\,\mathrm {S}$ and $5^{\circ }\,\,\mathrm {S}$, centred further from the Equator than the heating region. However, the pressure contour and flow pattern resulting from the idealised Matsuno–Gill model can be modified considerably by factors such as the shape and equatorial off-set of the heating region and the presence of background zonal wind^26^. In our case, the area of anomalous subsidence over South Amazon has a slight zonal off-set to the east of and is closer to the Equator than the heating in the Caribbean, similar to the results by Rodwell and Hoskins^23^.

To conclude, the seasonal cycles of North Atlantic tropical storms and South Amazon fires are in phase with a maximum around September and have significant inter-annual correlation driven exclusively by Caribbean tropical storms. Years of high fire activity are associated with atmospheric conditions over the Caribbean which favour tropical cyclones, enhanced precipitation, local ascent and remote anomalous Amazon subsidence. We hypothesise a direct physical link of tropical storms in the Caribbean to fires in the South Amazon through a thermal direct circulation, Hadley-type response driven by off-equatorial heating partly caused by the storms. Anomalous precipitation over the Caribbean regardless of cause releases latent heat there and contributes to the thermal direct circulation. However, Caribbean tropical storms play an important role based on our analysis as they account for much of the precipitation anomalies there. The anomalous Amazon subsidence we observe promotes favourable fire conditions there. Anthropogenic drivers, such as deforestation, are a major contributor to fires in the Amazon^8,28^. Some of the inter-annual variability is therefore due to the interaction of man-made burning with environmentally favourable conditions. By focusing on the fire season this study leads to a deeper understanding of Amazon fire variability than previous studies which considered the pre-season. This study helps build a more complete understanding of the drivers of Amazon fires and provides evidence of a case of remote physically linked compound extreme events.

## Methods

North Atlantic tropical cyclone data were obtained from NOAA’s IBTrACS Version 4^29,30^ for 1980–2019. Only cyclones with 1-min maximum sustained wind of $34\,\mathrm {kt}$ or above were considered. Precipitation data (2001–2018) were taken from NASA-JAXA’s TRMM 3B42 Daily^31^ and TRMM 3B43 (monthly)^32^ datasets. Fire data (2001–2018) were obtained from NASA’s MODIS Collection 6^33,34^. Fire pixels recorded in the MCD14ML product by the Terra satellite were filtered to keep only those classed as ‘presumed vegetation fire’ and with a detection confidence of at least $30\%$. These pixels were then adjusted for cloud coverage recorded in the MOD14CMQ product according to Giglio et al.^35^ The fire count over a region is the total number of cloud cover-adjusted pixels over that region. Zonal and meridional wind at $850\,\mathrm {hPa}$ and $200\,\mathrm {hPa}$, vertical velocity at $600\,\mathrm {hPa}$, SST, mean surface downward short-wave radiation flux and precipitation (1980–2019) data are the ECMWF’s ERA5 monthly averaged data^36,37^. The Canadian Forest Service Fire Weather Index Rating System (FWI) produced by the CEMS for the EFFIS^38^ gives the best dependency index skill score in South Amazon among the three indices studied by Di Giuseppe et al.^39^. The FWI depends on local noon relative humidity, temperature and wind speed among others.

To further understand the relationship between the North Atlantic and the Amazon, we focus on the following three regions: the South Amazon ($69^{\circ }\,\,\mathrm {W}$–$49^{\circ }\,\,\mathrm {W}$, $15^{\circ }\,\,\mathrm {S}$–$5^{\circ }\,\,\mathrm {S}$), which captures the most fire-active region of the Amazon (Fig. 1a); the August to October (ASO) Atlantic ITCZ ($48^{\circ }\,\,\mathrm {W}$–$17^{\circ }\,\,\mathrm {W}$, $2^{\circ }\,\,\mathrm {N}$–$13^{\circ }\,\,\mathrm {N}$), which captures the region of maximum precipitation over the North Atlantic in ASO; and the Caribbean ($88^{\circ }\,\,\mathrm {W}$–$52^{\circ }\,\,\mathrm {W}$, $10^{\circ }\,\,\mathrm {N}$–$25^{\circ }\,\,\mathrm {N}$), which covers the region with significant precipitation difference between the high and low Amazon fire years. We analyse the fire count, FWI, subsidence, surface solar radiation flux and precipitation over the Amazon; the tropical storm count and precipitation over the Caribbean; and the precipitation over the ASO Atlantic ITCZ. North Atlantic SST is averaged over the region $90^{\circ }\,\,\mathrm {W}$–$15^{\circ }\,\,\mathrm {W}$ and $0^{\circ }$–$25^{\circ }\,\,\mathrm {N}$. We also study the horizontal wind at $850\,\mathrm {hPa}$ and 850–200 hPa vertical wind shear over the North Atlantic for patterns favourable to the genesis and development of tropical cyclones. Composite differences over the periods from 2001 to 2018 and from 1980 to 2019 are taken between the top and bottom 5 and 13 extreme years respectively, corresponding roughly to the top and bottom terciles. The resulting anomalies are considered significant if their $95\%$ confidence intervals produced by bootstrapping do not bracket 0. The number of bootstrap resamples used for the maps and box averages are 1000 and 10000 respectively. All correlation coefficients are Pearson correlation coefficients. Unless otherwise specified, correlation analysis are performed on linearly detrended time series to eliminate long term effects such as global warming. This is achieved by subtracting the linear least squares fitted linear signal from the original time series. Non-detrended correlation results are reported only if the *p*-value indicates a change in the level of significance across the $99\%$ and $95\%$ confidence. We have identified all those cases clearly which move from significant to non-significant ($p > 0.05$). In those 3 cases the trends may be considered less robust.

All figures are generated using Python 3.8.3 with the library Matplotlib 3.3.4, and additionally the package cartopy 0.18.0 for maps.

## Data Availability

All data used in this study are publicly available. MODIS data are retrievable from the SFTP server https://www.fuoco.geog.umd.edu^34^. IBTrACS data is available at https://www.ncdc.noaa.gov/ibtracs/index.php?name=ib-v4-access. All other data can be obtained from the websites given in the relevant citations.

## Code availability

All codes produced to perform the calculations for this study are available from the corresponding author upon reasonable request.
